# Cyclodextrin-Based Polymeric Materials Bound to Corona Protein for Theranostic Applications

**DOI:** 10.3390/ijms232113505

**Published:** 2022-11-04

**Authors:** Donya Esmaeilpour, Jens Albert Broscheit, Sergey Shityakov

**Affiliations:** 1 Department of Chemistry, University of Isfahan, Isfahan 8174673441, Iran; 2 Center for Nanotechnology in Drug Delivery, School of Pharmacy, Shiraz University of Medical Science, Shiraz 713451583, Iran; 3 Department of Anesthesiology and Critical Care, University of Wuerzburg, Oberduerrbacher Str. 6, 97080 Wurzburg, Germany; 4 Laboratory of Chemoinformatics, Infochemistry Scientific Center, ITMO University, 191002 Saint-Petersburg, Russia

**Keywords:** cyclodextrin, theranostics, protein corona, nanomedicine, therapy, polymers

## Abstract

Cyclodextrins (CDs) are cyclic oligosaccharide structures that could be used for theranostic applications in personalized medicine. These compounds have been widely utilized not only for enhancing drug solubility, stability, and bioavailability but also for controlled and targeted delivery of small molecules. These compounds can be complexed with various biomolecules, such as peptides or proteins, via host-guest interactions. CDs are amphiphilic compounds with water-hating holes and water-absorbing surfaces. Architectures of CDs allow the drawing and preparation of CD-based polymers (CDbPs) with optimal pharmacokinetic and pharmacodynamic properties. These polymers can be cloaked with protein corona consisting of adsorbed plasma or extracellular proteins to improve nanoparticle biodistribution and half-life. Besides, CDs have become famous in applications ranging from biomedicine to environmental sciences. In this review, we emphasize ongoing research in biomedical fields using CD-based centered, pendant, and terminated polymers and their interactions with protein corona for theranostic applications. Overall, a perusal of information concerning this novel approach in biomedicine will help to implement this methodology based on host-guest interaction to improve therapeutic and diagnostic strategies.

## 1. Introduction

Cyclodextrins are supramolecular structures derived from potato starch by enzymatic transformation. These compounds were discovered in 1891 by Villiers [1]. They can improve the proficiencies of absorption, enhance solubility, modify the release kinetics of drugs and minimize side effects. The complexation capability of CD with the drugs can be specified by the binding constant (K) of the compound and the dissociation equilibrium of the complexation. The binding constant can be employed for evaluating the disassociation and association potency of the complex and the binding strength between the guest and host molecules. Additionally, the binding constant could be crucial for measuring the effect of CDs on in vivo absorption and bioavailability of drugs. Binding constants of host-guest interaction is an important parameter but frequently hard to measure using appropriate equipment [2].

**Figure 1:**
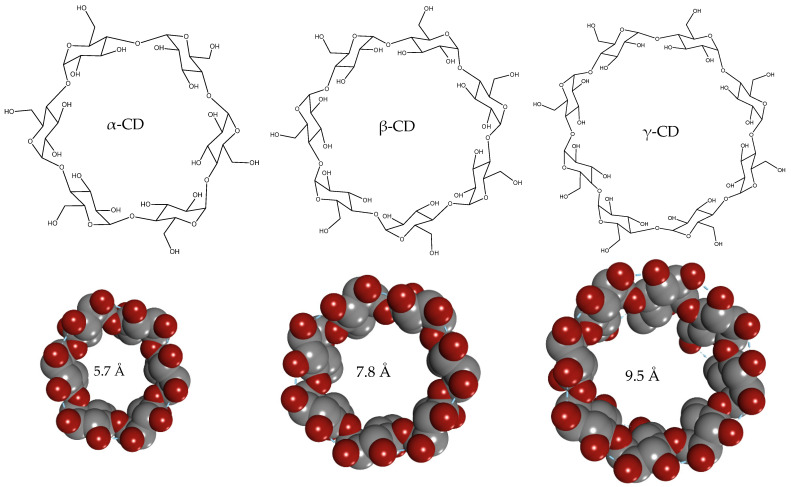
Structure and conformation of natural CDs.

CDs might be promising candidates for therapeutic and diagnostic purposes widely used in biomedicine. These structures can be chemically modified into branched star-copolymers forming various nanoparticles. Native CDs usually have primary and secondary faces where the hydroxyl groups are located. These faces are usually symmetrical and have different chemical properties creating torus-like molecules (Figure 1) [3]. Silveria et al. indicated that using CDs in high concentrations as nanoparticles might help to develop a construct (nanosphere) with hydrophobic sites for drug loading [4]. Besides that encapsulation, CDs can enhance the solubility of anticancer medicals, as determined by Correa et al., due to the improved drug stability. Significant attempts have also been made to produce CD-based polymeric materials [5,6].

The CD interaction with various drug-like molecules is mainly mediated by van der Waals forces facilitating host-guest inclusion of multi-layered particles. On the other hand, CDs could be organized in the form of star-shaped polymers creating condensed frameworks with critical arm densities, efficient synthetic pathways, and specific dynamic and rheological properties [7,8].

Some CD advantages over other polymers include biocompatibility, biodegradability, bioadhesive properties, and organ targeting, making them ideal for medical approaches and smart drug delivery. Therefore, in this review, we focus on recent cyclodextrin-based polymers, particularly polymers consisting of centered polymers, pendant polymers, and terminated polymers, in biomedical approaches. The main advances achieved with CD-based polymers (CDbPs) have been concentrated on reactive functional groups. CDbPs are categorized depending on their structural resemblances and specified CDbPs as CD-containing polymers or polymeric networks. This review discusses the following topics: (1) Practical implementations of CDbP, including rational drug design theory and structure-activity/property relationship (SAR and SPR). (2) Strengths and weaknesses of SAR/SPR related to the blood-brain barrier (BBB), theranostics, and regenerative medicine.

### 1.1. CD-Based Polymers for Theranostic and Biomedical Applications

As a new-emerged branch of biomedicine, theranostics is aimed at a combination of diagnostic and therapeutic strategies. Mainly focused on patient-centered care, theranostics offers a platform to move from conventional medicine toward more personalized and precise medicine [9]. The theranostics model exploits nanotechnology to combine diagnostic and medical features in a single approach. It includes new-generation targeting agents, imaging detection (MRI, ultrasound, and fluorescence), novel drug delivery (controlled release, anticancer, targeted release), genetic manipulations and modifications (gene knockdown and knockout), and treatment response monitoring [10]. Over recent decades, nanomedicine platforms have provided site-specific delivery and controlled release of cancer chemotherapeutics to improve the therapeutic efficacy with fewer undesirable side effects [11].

Therefore, nanoparticles as potential therapeutic agents can overcome distinct restrictions of conventional drug delivery systems (DDSs) like nano-specific targeting, poorly water-soluble drugs and oral bioavailability of peptides and proteins, and low therapeutic indices (TI < 0.50) [12,13]. The co-performance of therapeutic operations with personalized theranostic nanostructures can significantly advance personalized medicine and offer new advantages [14,15]. Theranostic nanomedicine can monitor drug delivery, release, and efficacy. Preliminarily, polymeric micelles, synthetic polymers, dendrimers, surfactant liposomes, and nanoparticles (solid or liquid) have been exploited as scaffolds for the physical encapsulation and covalent linking of anticancer drugs [16,17]. Nanomaterials-based biomedicine facilitates the synthesis of an innovative biocompatible and multifunctional material comprised of covalently cross-linked organic molecules. In particular, β-CD predecessors were cross-linked with inflexible organic linker molecules to receive tiny (~150 nm), temperature-stable, and extremely water-dispersible nanostructures with accessible pores [18]. CD are qualified candidates for theranostic applications due to their multifarious functional groups, enhanced flexibility, dependability, modular attribute, and biocompatibility that can be developed for numerous uses, particularly in ligand targeting [19,20].

Theranostics is the modern trend in biomedical science with tremendous potential for contemporaneous diagnosis and therapy for various diseases, such as cancer. Therefore, developing systems capable of early detection, reliable diagnosis, and beneficial therapy is critically important [21,22]. Yao et al. prepared stimuli-susceptible cyclodextrin-containing nanoplatforms for cancer-theranostic purposes [5]. Massoumi et al. provided novel multi-stimuli-responsive theranostic nanomedicines that were configured and manufactured by combining a thiol end-capped poly (N-isopropyl acrylamide-block-acrylic acid) (HS-PNIPAAm-b-PAA) onto Fe_3_O_4_@Au nanostructures and achieved physical loading of doxorubicin (DOX) as a common anticancer drug. They formed Fe_3_O_4_@AuNPs via a short Au nanolayer grown on larger magnetic NPs [23]. Similarly, Mingliang Pei et al. explored the cellular uptake of the DOX@β-CD-CQD theranostic agent by microphotography [24]. The authors hypothesized that DOX@β-CD-CQD could be incorporated into human hepatocellular carcinoma cells (HepG2) to release DOX [24].

Recent diagnostic improvements and minimal invasiveness of biological markers are aimed at resolving the challenges in cancer detection, care, and monitoring [25]. Early diagnosis of cancer can buy time and provide more effective treatment opportunities.

Cancer diagnostic approaches include physical examinations, laboratory tests (tests for tumor markers), imaging experiments such as magnetic resonance imaging (MRI), positron emission tomography (PET), and biopsy. The methodology is believed to be the best to diagnose cancer at an early stage. Cheng et al. developed an organic photodynamic therapy (PDT) technique derived from PEG-coated nano-micelles conjugated with 20% chlorine Ce6 (PEG-Ce 6 nano-micelles) to serve as an optical imaging agent. This PDT approach was successfully applied for PET fluorescence (PET/FL) dual-mode image-guided photodynamic anticancer treatments [26]. On the other hand, CD and their derivatives might be plausible candidates for cancer therapy and diagnostics using fluorescence imaging (FI), such as near-infrared fluorescence (NIRF). In addition, positron emission tomography/single-photon emission computed magnetic resonance imaging (MRI), tomography (PET/SPECT), photoacoustic imaging (PAI), and precision diagnostic imaging (PDI) were also integrated into nanomedicine [27,28,29,30].

Empirical evidence also demonstrates that nanoparticles that provide good therapeutic attributes may not necessarily be good diagnostic tools. As an illustration, some cancer detection protocols carried out by anti-EGFR-covered gold nanoparticles (NPs) of 20 nm exhibited strong cancer uptake. In contrast, NPs of 50 nm in size showed the highest contrast enhancement. This result highlighted that cancer theranostics might be size-dependent due to the restricted distribution of NPs in tumor tissue [31].

**Figure 2:**
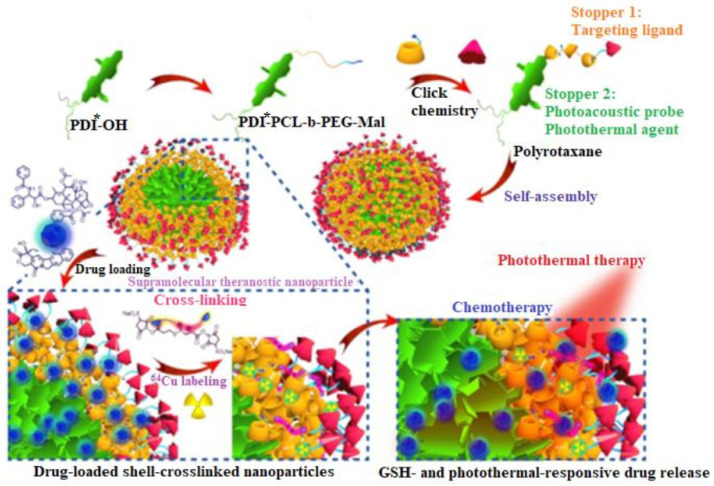
Diagram describes the production process of drug-loaded single-chain polymer nanoparticles (SCNPs) for dual-responsive drug release [38]. The abbreviations are PDI*: perylenediimide, PCL: polycaprolactone, PEG: polyethyleneglycol, and GSH: glutathione.

Clinically employed modalities of diagnostic especially computed tomography (CT), magnetic resonance imaging (MRI), and ultrasound (US) examination, anatomical information preparation under endogenous contrast. Moreover, NP-based contrast agents can increase in vivo imaging [32] by tuning NP size on CT [33]. Some in vitro and in vivo studies have shown associations between NP uptake [34] by tumor cells, biodistribution [35], and nanostructure size [36]. Mejia-Ariza et al. emphasized the use of supramolecular NPs, such as CDs, where particle formation, size control, and stability have governed by the nature of host-guest interactions [37]. However, the production of the polyrotaxane agent (PDI*-PCL-b-PEG-RGD β-CD-NH2) includes multiple stages (Figure 2), which might be considered a limitation. In particular, polyrotaxane could be employed as a theranostic pipeline, where the amphiphilic diblock copolymer acts as the axle and the primary-amino-containing β-cyclodextrin (β-CD-NH2) serves as the wheel, caused by the host-guest complexation between β-CD-NH2 and poly(ε-caprolactone) (PCL) segment (Figure 2) [38].

### 1.2. Significance of Protein Corona for Theranostic Applications

**Figure 3:**
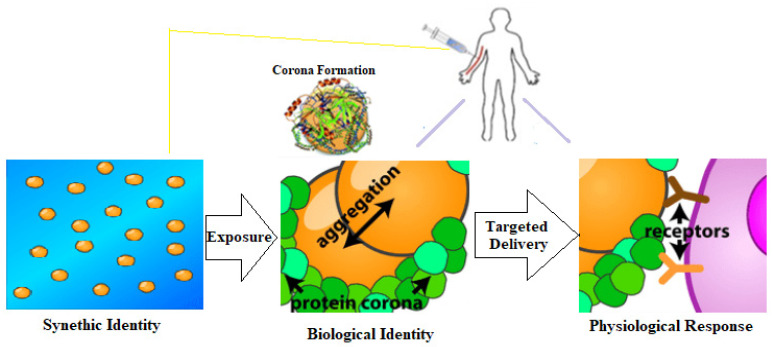
The interaction between protein corona, NP, and cellular receptors (physiological reactions). After NP injection into the blood, protein corona starts to aggregate at the NP surface as a part of biological identity. After that, cellular response can be triggered by specific protein receptors at the cell surface [44,45].

It is now recognized that when NPs come into contact with blood, proteins adsorb to their surface, creating what is known as a protein corona [39]. Protein coronas are very easy to form, altering the biological capabilities of nanosystems, particularly tissue accumulation, cellular uptake, antibody clearance, payload excretion profile, and toxicity. Therefore, these properties of protein corona could be harnessed to develop personalized remedies for every individual with high precision and speed [40,41]. Some biopolymeric-based nanoparticles might be useful for remediation treatment containing fatty acids, saccharides, or proteins. The process of protein corona formation around any nanosized particles into a framework has attracted more and more attention in recent years [42]. Hypothetically, protein coronas could also trigger changes in metabolic pathways, but these mechanisms are still poorly investigated. Many studies have also focused on the adsorption of polymeric nanostructures of proteins deposited around metallic NPs [43]. As such, the corona protein (biological identity) might aggregate over liposomes forming a synthetic construct (Figure 3) [44,45]. Therefore, understanding these phenomena at the molecular level could improve the diagnostic potential of existing and still-under-development diagnostic methods.

### 1.3. CD-Based Polymers Functionalized with Protein Corona at the Blood-Brain Barrier

Biological barriers represent defensive systems that protect important organs from various harmful effects of xenobiotics, poison molecules, and microorganisms (bacteria and viruses, etc.). Among them is the blood-brain barrier (BBB) that separates the blood and brain compartments. Due to this, CDs can adjust drug transport through the BBB boosting drug permeation via its passive diffusion mechanism [46,47,48,49,50]. Surface adsorption of NPs at BBB shows more intense fluorescence in the brain model than in other parts of the body.

Furthermore, due to the depleted biodistribution of quantum dots (QDs) and iron oxide-loaded polyethylene glycol succinate (PLA-TPGS) NPs throughout the BBB, less brain fluorescence intensity was observed [51]. To confirm this, a multimodal imaging system (MMIS) revealed sufficient NP penetration using highly sensitive consequential probes, allowing diagnosis to be achieved with each imaging [51,52].

Mousazadeh et al. determined that β-CD could increase the fluorescence of uncommon fluorescent polymers, where the polyamidoamine (PAMAM) and hyperbranched polyamidoamine (HPAA) variants were used [53]. Chen et al. demonstrated that the linkage of different amounts of β-CD onto the HPAA polymer for the constitution of HPAA–CDs hybrids increased the photoluminescence intensity and the limited molecular flexibility [54]. The steric hindrance model was developed using β-CD, where its rotation was restricted. On the other hand, the interaction of rhodamine B (RB) compounds through supramolecular interactions helped to form HPAA-CD@RB compounds. These molecules exhibited an enhanced fluorescence intensity due to increased amounts of β-CD. Furthermore, the increase in fluorescence of hyperbranched polysiloxane (HBPSi) was also confirmed by the group composition of biodegradable β-CD [55]. Recently, Lihua et al. produced HBPSi-CD polymer by attaching β-CD units to polysiloxanes to assess fluorescent properties [55]. The fluorescence emission from HBPSi-CD increased due to β-CD improving its stability and aggregation. Moreover, β-CD raised electron delocalization in HBPSi-CD aggregates, decreasing the HOMO-LUMO energy gap and leading to the red transmission/emission band [53]. Specific CDs and cell-permeable gene vectors have developed cellular internalization as CD cationic polymers and non-CD-containing macromolecular substitutes.

Some results have been demonstrated to formulate small interfering RNA (siRNA) nano-assemblies using cationic diphenylalanine (CDP), CD-polyrotaxanes (PRs), CD-dendrimers, and CD-targeting ligands [56]. Chemical modification of CDs, usually by altering their surface charge, reduces the effectiveness of protein repellents, whereas their size reduction produces their accumulation in the lung or kidney and other tissues significantly [56].

On the other hand, PRs might be among the most promising analogs to facilitate interactions between anionic genes and cationic polymers due to the convenient manipulation of cyclic macromolecules attached to axial polymer chains [21]. Biodegradable PRs containing polyethylene glycol (PEG) polymer chains and various α-CDs have shown excellent potential in drug delivery methods [21]. The implementation of CD-based PR can be made more specific by adapting the PR end-to-end to include fluorescent-detectable segments for special applications. Moreover, cleavable cationic PRs can enhance target gene silencing through the efficient delivery of therapeutic siRNAs [21].

## 2. Cyclodextrin-Based Polymers

### 2.1. Structural Features of Cyclodextrin-Based Polymers

**Figure 4:**
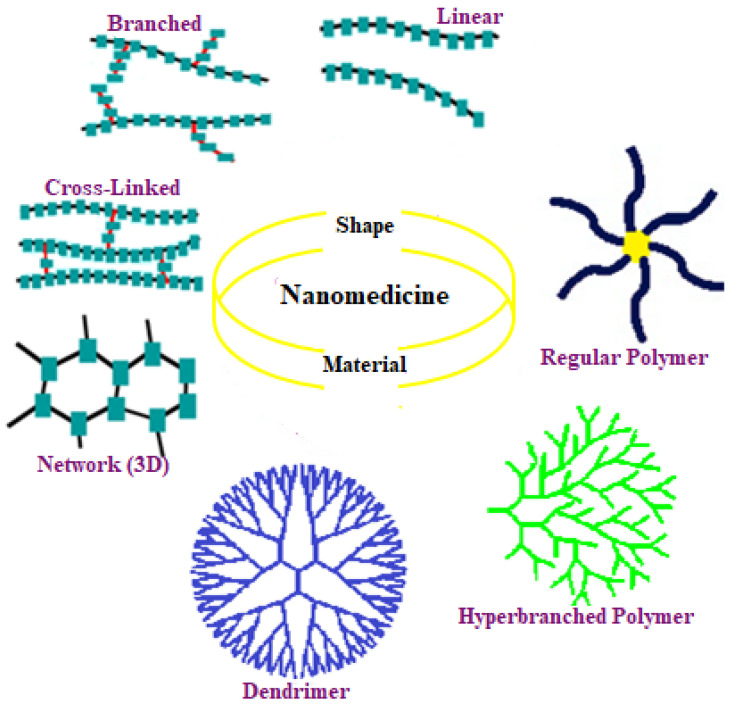
Classification of polymers by molecular topology.

CDbPs are various polymers containing CD-based moieties to improve polymer physicochemical characteristics and functions. The physical properties of a polymer mainly depend on its molecular chain structure giving rise to four categories: linear, branched, cross-linked, and topologic (Figure 4) [57,58]. The basic difference between linear and cross-linked polymers is that linear polymers are straight, whereas cross-linked ones have branched molecular topology possessing unique spatial features.

At the beginning of the 1950s, Flory developed a hypothesis to describe the polymer network and the associations between the topology and mechanical properties such as elasticity [59]. In the 1980s, Duplantier developed a hypothesis to demonstrate any polymer network topologies using statistical mechanics, which could assist in determining topology-dependent crucial agents in a polymer network [60]. In the early 2000s, Tezuka and colleagues were the first research group who consistently demonstrated a single molecular chain with topological details [61]. Indeed, supramolecular polymeric molecules would have been regarded to be favorable carriers for biomedical and theranostic applications in cancer and cardiovascular research [62,63].

Due to their large amounts of hydroxyl groups on their external surfaces and hydrophobic cavities, CDs can link to different positions of the polymers. Recently, several interesting CD-based polymeric systems have been developed [64]. The latest progressions in the controlled/living polymerization approaches combined with the highly efficient coupling reactions have offered feasible synthesis methods to prepare complex dendritic polymer structures. These polymers are highly branched as a 3-D network consisting of at least several subclasses [64]. Now, the CDbPs substances can be categorized depending on their structures: (1) CD-terminated polymers (CDTP), which have two CDs at their ends. To achieve CD-terminated polymers, the homo/block copolymers can be instantly grown from a CD-based large initiator or can be affiliated with a CD-based derivative after polymerization [65,66]; (2) CD-pendant polymers (CDPP), in which they can be classified into two categories. One is accomplished by a replacement reaction between CD derivatives and the target polymers [67,68,69]. The other is produced by the straight polymerization of CD-based monomers alone or with other monomers [70]. Polymers with pendant CD categories on the side chains often possess macromolecular hosts with numerous binding sites. Host-guest polymer assemblies across nano, micro, and macro scales originating from these CD-pendant polymers are attracting more and more interest in nanostructure fabrication, pharmaceutics, and biomedicine [50,71]. CD-centered core polymers (CDCCP), in which CDs function as the core for forming multi-arm polymers, could also be used for rational drug design and discovery [72,73,74]. Due to advances in polymeric science, CDs can be characterized as biomedical agents in these hybrid polymeric systems.

#### 2.1.1. Cyclodextrin-Centred Core polymers

Star polymers are composed of several centrally attached linear polymers with many chains and functional groups. The polymer core or center can be atoms, molecules, or macromolecules and chains or arms composed of organic chains of different lengths. Synthesis of star polymers using inexpensive and functional CDs has advantages such as cost efficiency, ease of manipulation, and easy access to multiple functions. Due to the relative order in which the polymer composite arms are made, the synthesis of star polymers using CD cores can be divided into two categories: core-first and arm-first approaches. The core-first approach uses CD-based initiators to grow polymer chains directly synthesizing CD-centric core star polymers. For the arm-first approach, a conjugation reaction is used to synthesize a CD-centred core star polymer, and the synthesized linear polymer is attached to a CD derivative. The advantage of the second method over the previous one is that each arm has the same chain length [5]. As unique structures, star polymers exhibit unique properties that simple linear polymers cannot match. Star polymers are an important group of technological nanostructures used or studied in various nanotechnology applications such as theranostics. The most basic form of the branched polymer is the star-shaped polymer first introduced by Schaefgen and Flory in 1948, who synthesized multiple polymer chains in a star-shaped polyamide [75,76]. A revolutionary breakthrough in star polymers was reported in 1962 by Maurice Morton et al. [77] as they developed the first method of synthesis of the famous star-shaped polymer by anionic polymerization. To date, many studies have focused on the properties, synthesis, and function of star polymers [78].

In this way, linear polymers can be easily defined by obtaining a specific analysis of the arms. Identifying specific compounds in CD-centred core star polymers is a major challenge, as abundant linear polymers need to be increased by enlarging all active sites of cyclodextrins. To achieve high purity for star polymers with CD-centric cores, free linear polymers should be eliminated, often requiring tedious purification processes.

#### 2.1.2. Cyclodextrin-(Pendant and Terminated) Polymers

CD-capped polymers can be obtained by placing a CD at one or more termini of the polymer. CD-terminated polymers can be synthesized directly using CD-based macro-initiators. These polymers can also be obtained using linear polymer linkages with reactive sites on functionalized CDs.

The CD side-chain polymers are synthesized in two ways: by direct polymerization of a large number of CD monomers or by functionalization of CDs onto the side chains of the polymer. Although it is difficult to synthesize supramolecular polymers with complex structures by conventional covalent routes, these compounds exhibit host-guest interactions in which a polymer with CD side chains and a polymer with a CD terminus act as a host can be obtained [79]. Finally, recent advances in cyclodextrin self-assembly provide the means to design diverse nanomaterials, such as Vesicles, micelles, nanogels, supramolecular hydrogels, complex superstructures, and multifunctional structures, offer a simple combinatorial approach [62,80].

For example, important polymers synthesized as building blocks for CDs include star polymers, polyrotaxanes (PR), supramolecular block copolymers (BCPs), and comb polymers [81]. These CD polymers could be used for drug loading and controlled release via supramolecular inclusions. The hydroxyl groups of these compounds also participate in polymerization and bioconjugation reactions. Several cross-linkers have also been used for CD polymerization, notably epichlorohydrin, ethylene glycol digital ethers, isocyanates, and polycarboxylic acids [81].

### 2.2. Application of Cyclodextrin-Based Polymers in Theranostic Nanomedicine

Key polymers, such as CD-centric core polymers, CD-side polymers, and CD-terminated polymers, have a variety of applications, including materials fabrication for theranostic nanomedicine. Due to this, cyclodextrin-based polymers have been validated as effective scaffolds in nanomedicine [82,83].

CD-based polymers are an attractive platform for theranostic nanomedicine (Table 1). It is well known that many drug-like molecules have insufficient water solubility. Traditional formulations of insoluble drugs involve a combination of bio-based solvents, surfactants, and extreme pH conditions that often lead to unfavorable and adverse reactions [84]. Therefore, safe CDs might be a perfect choice to stabilize the active pharmaceutical ingredients to reduce volatility, cytotoxicity, or bitterness. For instance, CD-drug formulations can improve not only the water solubility of many poorly water-soluble substances but also their bioavailability and therapeutic efficacy.

The first method for preparing high molecular weight (Mw = 104) water-soluble polymers was described by Solms and Egli in 1965 [85].

Industrial α-, β-, and γ-CD polymers have been investigated for use as eluents in adsorption chromatography (on cellulose thin films) using aqueous solvents. Lederer et al. developed an approach to improve a polymer elution compared to α-CD [86]. Various cleansing active ingredients (especially methylene blue (MB), ethacridine lactate (acrinol), brilliant green (BG), gentian violet (GV), fuchsin acid (FA), cetylpyridinium chloride (CPC)) in CD-based polymers were tested to treat of foot ulcers, infected wounds, and burns [87,88]. The eluents usually contain soluble polymers, while the CD polymers for wound treatments are water-insoluble ones. Therefore, the swelling effect of CD polymers might be an important advantage in wound treatment.

Hyperbranched polymers (HBPs) and hyperbranched polyglycerols (RPGs) are among many dendritic structures that combine excellent functionalization capabilities and stability with low viscosity. Therefore, RPGs are considered promising for theranostic nanomedicine applications for cancer treatment [89].

The rheological properties of star polymers are also important compared to linear polymers. These polymers have a low intrinsic viscosity, a small hydrodynamic radius, a large critical micelle concentration (CMC), and, therefore, low aggregation. Intrinsic viscosity increases with increasing branching functionality and molecular weight. Once the functional efficiency saturates, the viscosity limitation is related only to the molecular weight of the arms. This may be attributed to increased repulsive interactions due to more heterocontacts between different arms [90].

Tang et al. prepared star-shaped polymers via ring-opening polymerization (ROP) followed by reversible addition-fragmentation chain transfer polymerization (RAFT). In this experiment, DOX was used as a standard drug to conjugate the copolymers via an acid-labile hydrazone bond. Analysis of the solution properties of as-prepared DOX conjugates is greatly influenced by the composition and different structures of the polymers. Among the conjugates studied, an eight-arm triblock star polymer related to poly (ethylene glycol) (PEG) and N-2-hydroxyl methacrylamide (PHPMA) showed a poor cell penetration and fragmentary colocalization within lysosomes [91].

A particular star-shaped amphiphilic polymer consisting of a β-CD core with hydrophobic (lactic acid) (PLA) and hydrophilic (ethylene glycol) (PEG) poly arms was prepared for the delivery of anticancer drugs [92].

The study revealed that this star-shaped single-molecule micelle generated from β-CD-PLA-PEG was more efficient than Genexol-PM for cancer treatment [92,93].

Namgung et al. reported a novel nano-assembled drug delivery device fabricated by multifunctional host-visitor interactions between polymer-cyclodextrin and polymer-paclitaxel (PTX) complexes [94].

Furthermore, Badwaik illustrated the production of three Pluronic^®^-based, cholesterol end-capped cationic polyrotaxanes (PR+) mixed with 2-hydroxypropyl-β-cyclodextrin (HPβCD) for small interfering RNA (siRNA) delivery. The data confirmed 90% of cell viability and a significant silencing effect (>80%) in cell lines such as HeLa-GFP and NIH 3T3-GFP [95]. Gomez-Garcia et al. carried out a manufacturing technique to develop a library of clear-cut multivalent glycoclusters with a showcase of α-mannosyl (α-Man) and β-lactocyl (β-final) antennas using β-CD [96]. Albuzat et al. exhibited that the polyrotaxane PRx2 assembled from a hexatonic α-CD derivative 1 and a cationic polymer I-11 is highly compatible with three cell lines. Additionally, this compound possessed improved transfection potencies in vitro. The molecular mechanism behind it could be explained by the steric hindrance of Coulomb interactions exerted by the natural α-CD rings [97].

**Table 1:** Cyclodextrin-based polymers for theranostic nanomedicine techniques.

Polymer	CD Nature	References
Poly-paclitaxel	β-CD	[80]
PEG-PPG-PEG polyrotaxane vectors	2-HP-β-CD	[81]
Cationic cyclodextrin polyrotaxane	α-CD	[83]
Cyclodextrin polyrotaxane	CD	[85]
Hyperbranched polyglycerol	β-CD	[95]
Poly(glycidyl methacrylate)	β-CD	[93]
AIE-active dye with β-cyclodextrin terminated polymers	β-CD	[86]
An-HPG-βCD	β-CD	[87]
Epsilon-polylysine-grafted-PEI-βCD	βCD	[90]

Arima et al. demonstrated the gene transfer efficiency of PAMAM starburst dendrimer generation 2 (G2) coupled with α-CD (α-CDE conjugate (G2)). It has been found that lactose-tagged α-CDE [Lac-α-CDE (G2)] can be a promising tool for gene and siRNA delivery [98].

Dandekar et al. scrutinized the capability of a prepared cationic polyrotaxane for siRNA binding into nanosized compounds via transfection of siRNAs samples targeting nucleotides of firefly pGL3 luciferase (Luc siRNA) to develop small interfering RNA molecule for curative approaches [99]. In cellular experiments, the polymer increased the knockdown of a gene method required in tuberculosis pathogenesis when the nano-conjugates appeared in contrast to free siRNA. The strength and cellular non-toxicity of cyclodextrin-polyrotaxane further detailed clarification in vivo testing of tuberculosis (TB) and other intracellular bacterial infection samples, which facilitate further consideration of their clinical potential [99].

Moreover, the hydrophobic tetraphenylethylene (TPE)-AIE (aggregation-induced emission)-active dye (TPE-Ad) was encapsulated inside a hydrophobic core, and the hydrophilic polymer covered the surface of the hydrophobic core and acted as a shell-forming material [100]. TPE-β-CD-PEG copolymers were determined by many types of instruments. The cytocompatibility and cellular uptake behavior of TPE-β-CD-PEG were also considered to evaluate potential biomedical applications. The results showed that TPE-β-CD-PEG copolymers tended to self-assemble into luminescent nanoparticles that exhibited remarkable water-dispersibility, AIE properties, and excellent biocompatibility. Due to this property, TPE-β-CD-PEG has great potential for biomedical applications. Compared to conventional techniques that provide AIE-active copolymers, this technique is very simple for the following reasons: easy handling (stirring at room temperature without adding catalyst), high atomic economy, short preparation times (<30 s), and high yields. With all such advantages of this technology, conjugating AIE dyes to PEG using β-CD as a bridge is an innovative idea, allowing various multifunctional AIE-activated polymer conjugates for numerous implementations. It was predicted to be a popular technique for further developing the body [100].

Huang et al. suggested AIE-lively composites (An-HPG-β-CD) configurations with tested dazzling fluorescence capabilities, length of the uniform and small particle, and incredible biocompatibility were first fabricated through the constitution of dynamic phenyl borate among An-B(OH)_2_ (9, 10-bis (divinylphenylboronic acid) anthracene) and hyper branched polyglycerol functionalized beta-cyclodextrin (HPG-β-CD). In evaluating commonplace strategies for preparing AIE-active polymers, this strategy only specifies facile reaction circumstances at room temperature (RT), freed from catalysts and air surroundings. The An-HPG-β-CD configurations might self-gather into middle-shell micelles in an aqueous solution because of the amphiphilicity. The hydrophobic An-B(OH)_2_ become accumulated within the center and emitted brilliant Yellow fluorescent protein (YFP) because of its attribute of aggregation-induced emission (AIE). Hydrophilic HPG-β-CD will coat the floor of the supramolecular and form the shell to render them water dispersion. Most crucially, the holes of β-CD in An-HPG-β-CD compounds allow the encapsulation of several organic molecules. Therefore, it is emphasized to consider their biological imaging and drug shipping utilization. Given their enormous potential, it is thought the An-HPG-β-CD configurations have bright prospects in biomedical applications [101].

Matyjaszewski et al. reported on the current status and prospects of atom transfer radical polymerization (ATRP). ATRP is a tremendously vigorous and multifaceted manufactured methodology for developing polymers with a controlled and site-specific functionality. The essentiality of a meticulous synthesis is a precise mechanistic comprehension of the ATRP technique via correlations of comprehensive system-reactivity comprising the influence of monomers, polymerization initiators, ligands, additives, solvent, temperature, and pressure molecular dynamics technique Copolymers enabled by ATRP, and hybrid materials suggest uses. These hybrids might be derived from polymers formed by ATRP covalently bound to other polymers provided by other techniques, such as polycondensation (polyethers, polysulfones (PSF), polythiophenes (LCPs)), coordination polymerization (polyolefins), anionic polymerization (polyenes), cationic polymerization (polyisobutene (PIB)), ring-opening polymerization (ROP) (polyethers, polyesters, polyoxazolines (POx), or polycycloolefins [102].

Chen et al. reported a new synthesis of amphiphilic fluorescent copolymers via host-guest interaction under β-CD-based polymers and aggregation-induced emission (AIE) active dye (Ad-PhNH2). These polymers were prepared for the free radical polymerization and reaction of subsequent ring-opening. The polymeric AIE active drug delivery system was prepared for the subsequent ring-opening reaction and free radical polymerization as well. The polymeric AIE active drug delivery system can self-assemble into Fentanyl Pectin Nasal Spray (FPNS) because of amphiphilic architecture. The hydrophobic dye accumulated in the core, and, therefore, can discharge high fluorescence intensity due to its aggregation-induced emission (AIE) attribute. But the hydrophilic polymers coated on the hydrophobic core can enhance adequate dispersibility in pure aqueous solution. Physiological consideration consequences illustrated that PEGMA-IA-β-CD/Ad-PhNH2 FPNs could be successfully internalized into cells and manifest little cytotoxicity. Most essentially, the molar ratio of β-CD to Ad-PhNH2 can be effortlessly adjusted, and the surplus β-CD can be utilized for carrying anticancer factors. An overwhelming number of carboxyl groups were produced by the ring-opening reaction. The indigenous carboxyl residues might improve conjunction reaction and natural delivery. The attribute of PEGMA-IA-β-CD/Ad-PhNH2 FPNs makes these compounds appear in biological imaging and delivery systems for biomedical applications [103].

Sukumar et al. showed the potential of the proficient nose-to-brain direct transport route to the strategy of bypassing the blood-brain barrier. It also made it viable for centered transport of the theranostic polyfunctional gold-iron oxide nanoparticles (polyGIONs) level supplied with load medicinal therapeutic miRNA (miR-100 and antimiR-21) to glioblastoma (GBM) in mice. As a result, the nanoproduct could enable the demonstration of GBM cells with the methodically transported chemotherapy drug temozolomide (TMZ). Over and above, the delivery of nanoparticles for multimodal molecular and anatomic imaging in vivo studies, drug trafficking, and curative impacts. Initially, they prepared GIONs covered by β-CD/CS hybrid polymer and co-loaded with miR-100 and antimiR-21. Following this, they adorned their surface with PEG-T7 peptide utilizing host-guest chemistry of CD-adamantane. The consequential (resultant) polyGIONs illustrated green micro RNA (miRNA) loading with the raised balance of serum. They characterized them for particle length, polydispersity index (PDI), polymer functionalization, transmission electron microscopy (TEM), and quantitative reverse transcription PCR (qRT-PCR). For in vivo intranasal shipping, they used U87-MG GBM mobile-miRNAs in mice treated with T7-centred polyGIONs, as explored by in vivo optical fluorescence and magnetic resonance (MR) imaging [104].

Yao et al. reviewed the current advances in the design, synthesis, and application of CD-based copolymers, also approaching applications of CD-based polymer materials, for instance, hydrogels as intelligent material, synthesis of an inorganic substance, cancer imaging, and self-assembled nanomechanics [105].

Zhou et al. reported a highly efficient drug/gene co-delivery system (PCL-HPG-PE1600). This is an oligoethylenimine-linked to β-CD (β-CD-PE1600), and a Benz imidazole-modified four-arm polycaprolactone-initiated hyperbranched polyglycerol (PCL-HPG-BM) loaded with DOX and matrix metalloproteinases-9 (pMMP-9), based on supramolecular inclusion complexes. Supramolecular chemistry PCL-HPG-PEI600 showed adequate delivery of DOX and pMMP-9 and high pH sensitivity. Upon DOX release, PCL-HPG-PEI600/DOX exhibited pH-controlled DOX release and concentration-dependent inhibitory effects on MCF-7 cell lines. Regarding matrix metalloproteinase 9 delivery, PCL-HPG-PEI600 showed superior pMMP-9 delivery, significantly affecting greater transfection efficiency than in vitro/in vivo measurements of PEI25k. Overall, gene-presenting drug delivery devices for alternative therapies have emerged as promising techniques for cancer therapy. Although the impact of methodologically developed in vivo therapies is driven by their strong delivery capacity, low toxicity, and adequate stability in blood, problems remain. To reach the goal, among others, supramolecular investigations are carried out to establish drug–gene co-delivery systems with superior capabilities [106].

### 2.3. Application of Cyclodextrin-Protein Corona in Biomedical Approaches

The formation of protein coronas on the surface of nanosized particles endows nanosized particles or polymer systems with novel biological properties, suggesting numerous applications in biomedicine or theranostic nanomedicine. The following subtitles embody the latest advances in nanosized particles and protein corona structures for biotechnological applications.

Yallapu et al. have investigated the sample of human serum protein corona (HSPC) formation with nanosized particles. The change in particle size, zeta potential, hemotoxicity, cellular uptake/cancer cells targeting potential, and MRI characteristics of nanosized magnetic particles were reduced because of the adsorption of human serum. Meanwhile, associated with raised serum and particle concentrations, Apolipoprotein E (APOE = gene, apoE = protein) is absorbed in the surface of nanosized magnetic particles besides serum albumin (HAS) and transferrin (TF). But there existed no conspicuous primary-secondary structural developments noticed in serum proteins by various approaches, including Fourier-transform infrared spectroscopy (FTIR), X-ray diffraction (XRD), and circular dichroism (CDS). Hemolysis evaluation offers nearly no hemolysis at the approved concentrations (up to 1 mg/mL) for nanosized magnetic particles compared with the sodium dodecyl sulfate (positive control). Moreover, the developed internalization and uptake of magnetic nanoparticles through the C4-2B cell line appears to have been confirmed during incubation with human serum (HS). Following the adsorption of serum protein to the surface of nanosized magnetic particles, the immediate proximity within T1 (~1.33–1.73 s) and T2(~12.35–13.43 ms) times of relaxation permitted nanosized magnetic particles to maintain intrinsic magnetic resonance imaging potential even after adsorption of biomolecular protein. All high-level clinical parameters could dictate the translation to clinical practice and the use of this formulation for next-generation nanoscience for drug delivery (DD), cancer targeting, imaging, and theranostics applications [107].

Polymer-based nanocarriers are particularly useful as they offer a high degree of compatibility, and Fluorescence durability correlation spectroscopy (FCS) methods are powerful and functional tools for NCs (nanocarriers) at all levels of nanotechnology processes. In particular, fluorescence correlation spectroscopy (FSC) has been used to investigate the size of NCs and the medical efficacy of post-development loading, the stability of NCs, and possible mediation between the plasmatic and vascular flow. You can run and compute things of drug release in the cytoplasm of target cells [108].

### 2.4. Mechanism of Protein Fibrillation

**Figure 5:**
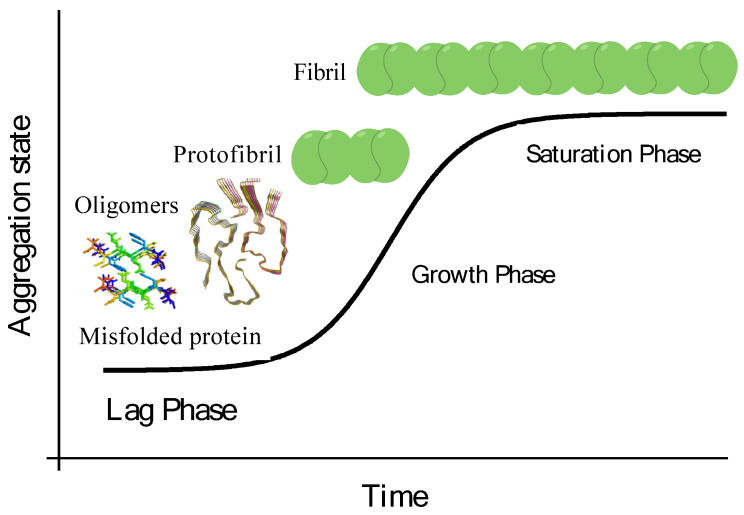
The kinetic proceeding of protein amyloid fibrillation comprising (Lag phase) aggregation of misfolded monomers into tiny intermediate oligomers; (Growth phase) re-arrangement of these oligomers into organized fibrils containing the cross-beta structure; (Saturation phase) association of beta structured oligomers into proto-fibrils [120].

Throughout the first phase, monomers self-associate to form cores or oligomers. Whenever a critical core size is reached, the second stage (the elongation stage) relies on an exponential function around the fibril by successive additions of monomers. At this level, formed protofibrils assemble into fibrils and finally into insoluble mature fibrils (Figure 5). Although amyloid fibrils are generally thermodynamically stable, these structures exist in equilibrium between monomeric and oligomeric species [109]. Amyloid aggregation appears to be lineage-specific and may involve similar hydrophobic proteins or different proteins [110]. Protein ongoing amyloid fibrillation and deposition in tissue are discovered in conformational disorders, proteinopathies, or protein misfolding disorders (PMDs) like Alzheimer’s (AD), Parkinson’s (PD), Huntington’s (HD), amyotrophic lateral sclerosis (ALS), and Creutzfeld-Jakob disease (CJD), and so forth. The particular mechanisms at the rear of the fibrillation approach remain elusive, and there is still an absence of peculiar restorative manners for protein fibrillation-related diseases. Researchers are still faced with difficulties in discovering the mechanisms of fibrillation formation and using powerful strategies to inhibit fibrillation in the human body. The particular introduction of nano-scaled materials (NSMs) in nanotechnology applications medicine provides an opportunity to address the existing challenges, as NSMs have been suggested that stop the procedure of fibrillation to a distinct degree [111,112].

Amyloid beta fibrils (AβF) are discovered in patients with Alzheimer’s disease (AD). The amino acid sequence of 17–24, 30–36, and 38–42 are investigated as hot spot residues of the Aβ peptide and get broadly determined as induce factors for the fibrillation procedure. Bare AuNPs illustrated to reduce the rate of the fibrillation method of Aβ1-42 is impeded by adsorption of the hot spot residue 17–24 to the having a positive charge AuNPs [113]. The inhibitory activities of bare nanoparticles (NPs) were also verified to be dose-dependent. That is, a dense concentration of NPs suggests a greater approachable surface for trapping higher amounts of Aβ1-42 monomers than a minor concentration of NPs, leading to enhanced inhibition activity. Protein corona-coated nanoparticles illustrate fewer inhibitory impacts on fibrillation. PCs give a negatively charged surface on the NPs, and the negatively charged parts of the hot spot residue [114] exhibit less binding tendency of corona-coated nanoparticles, bringing about a reduction of the surfaces of the nanoparticles tend to the Amyloid β (Aβ) monomers. Consequently, protein corona-coated nanoparticles possess less opportunity to trap Amyloid β monomers, assisting the self-assembly of considerable Amyloid β (Aβ) monomers residual in an aqueous solution. Over and above that, the inhibitory activities of corona-coated nanoparticles were confirmed to be protein source and concentration-dependent due to distinctions in the clotting factors of proteins. This demonstrates that a fetal bovine serum (FBS) corona presents a higher inhibitory impact on NPs regarding Amyloid β fibrillation than human plasma (HP) corona, whereas nanostructures with protein corona illustrate a lower inhibitory impact than those from 10% HP/FBS. A thorough study has been carried out on Aβ25-35 with human plasma (HP) or cerebrospinal fluid (CSF) coronas [114]. These inhibitory effects are believed to be related to the Aβ peptide and protein corona. Usually, due to the large capacity for capturing Aβ1-42 monomers, bare NPs represent remarkable inhibitory effects on the Aβ1-42 fibrillation proceeding. Compared to plasma-coated NPs, the NPs surfaces, nevertheless, are not covered by the CSF model. Therefore, CSF-coated NPs have more approachable surfaces for capturing Aβ1-42 monomers and blocking the aggregation of hot spot residues. Hence, plasma-coated gold nanoparticles (AuNPs) and gold nanorods (AuNRs) show less inhibitory activity on the Aβ1-42 fibrillation kinetics than CSF-coated or pristine AuNPs or AuNRs. Concerning the Aβ1-42 peptide, pristine nanostructure accelerates its fibrillation proceeding, while corona-coated nanostructures inhibit the fibrillation proceeding. Multiple hydrophilic residues in the N-terminal (25–30) of Aβ1-42 exist, and multiple hydrophobic amino acids are in the hot spot residue (30–35). The hydrophilic NPs prefer to bind to the hydrophilic region instead of the hot spot residue; Aβ1-42 monomers accumulate within the nanostructured surfaces and have the propensity to form fibrils led by the unbound hot spot residue. Also, to amyloid β peptide, an influence of the nanostructure-protein corona compound on the amyloid fibrillation development of human islet amyloid polypeptide, the fibrillation of which is a hallmark of type 2 diabetes (T2Ds) was further reported [115]. The β-lactoglobulin (BLG) protein is a natural β-sheet-rich protein; its architecture mainly turns into an α-helix after heat treatment. Suggesting BLG and heat-denatured BLG-coated AuNPs to IAPP confirms intercalation of BLG-AuNPs with IAPP is led by β-sheet stacking. The fibrillation development of IAPP can be led to BLG-coated AuNPs since the interactions among the BLG-AuNPs and LAPP block the IAPP self-assembly. It prepares novel evidence for the detection and inhibition of protein fibrillation.

Lotfabadi et al. supplied innovative ideas for surmounting issues and restrictions associated with traditional therapeutics approaches to suggest the expectancy of patients.

Shinde et al. demonstrated a supramolecular approach to successfully inhibit and/or degrade amyloid fibrils generated from human insulin and normal lysozyme proteins by processing SBE7β-CD macrocycles. Sulfobutyl ether-β-cyclodextrin (SBE7β-CD), a water-soluble macrocycle, is probably a successful additive that inhibits fibril development and breaks mature fibrils into small, non-toxic allergens. Steady-state expression and time-resolved fluorescence, circular dichroism ratios, and fluorescence micrographs collectively describe the inhibition and disruption of amyloid fibrils in the region of SBE7β-CD. Macrocyclic encapsulation of specific amino acid residues on proteins balances the native forms of insulin and lysozyme, preventing conversion to β-sheet conformers and thereby inhibiting fibrosis. However, the overall positive charge on the fibril surface and negative sites on the SBE7β-CD host made disaggregation of the fibril population achievable. On the positive side, the non-toxic SBE7β-CD component reduces system toxicity and holds promise as a therapeutic agent for amyloidosis. Further investigators are finalizing various protein systems/clinical selections to evaluate the efficacy of SBE7β-CD in cell culture media [116].

On the other hand, some studies indicated the successful inhibition of Aβ fibrillation by CD-polymer conjugates [117]. In particular, the poly-fluorene-alt-benzothiadiazole loaded with CDs was used to recognize and remove Aβ fibrils to efficiently treat AD. Furthermore, 5[4-(6-O-β-cyclodextrin)-phenyl],10,15,20-tri(4-hydroxyphenyl)-porphyrin and its zinc complex were tested to interact with Aβ and inhibit its fibrillar aggregates [118].

Typically, fluorescence (FL), circular dichroism and *Fourier-transform infrared spectroscopy* (FTIR) spectroscopy, and *atomic force microscopy* (*AFM*) divulge that both approaches to the peptide stored its fibrillation attributes and organized fibrils. Yet, the combined fibrils organized more swiftly than the free peptide, and were long and thin rather than the thick and twisted morphology of the intact peptide. Thus, the limitations presented by the scaffold regulate the structure of the fibrils but do not obstruct the actual fibrillation process. Exploiting fibril-forming peptides (FFPs) in the design of nanomaterial remained a challenge due to the problems in assessing and controlling how amyloid fibrils form (Table 2) [119].

The particular idea of protein corona adsorption adds to new techniques for dwindling potential nano hazards and for using a more logical design of nanoplatforms. It also is a principal program and mechanism for nanostructures to provoke protection from infection. The brand-new fundamental natures are prognosticated to increase the efficacy and bioavailability of nanostructures as possible drug delivery and theranostic factors.

## 3. Conclusions and Future Perspectives

While polymeric materials (PMs) and nanocarriers (NCs) are introduced into a biological milieu, the protein-adsorbed PMs and NCs, as opposed to the bare PMs and NCs, indicate the real identities and therapeutic responses of the PMs and NCs. An improved understanding of PM-PC and NC-PC compounds is an extraordinarily significant opportunity to improve novel nanomaterials for developing their approachability to medical science and theranostic nanomedicine.

The main advances of CDbPs, such as CD-centred core, CD-pendant, and CD-terminated polymers, are reactive functional groups. Typically, the great attributes of both cyclodextrin and nanotechnology systems will simultaneously suggest extra avenues for cancer treatment strongly. In addition, we have a prerequisite to creating usage of the efficiency of these CD-based nanocarriers utilizing in vitro models for targeted tumor therapy and the degree of toxicological tests of cancers, namely life-threatening diseases. CDbPs bring new opportunities for the arrangement and preparation of new functional nanostructured materials. CDs provide a wide range of derivatives that can be employed in developing moiety diagnostic and therapeutic agents through chemical combination and cross-linking interactions. Ultimately, CDs could be used to customize powerful anticancer drugs to bring about impressive treatment. In addition, the protein corona specifies the destiny of polysaccharide nanoparticles (PNPs), and they play a key role in nanotechnology, in particular, in theranostic nanomedicine. Thus, derivatives of CD such as the randomly methylated-β-CD, sulfobutylether-β-CD, glucosyl-β-CD, and poly (ethylene glycol)-CD copolymers were synthesized to realize the necessity for in vivo studies.

Even though various endeavors appeared constructed to distinguish CD-PMs-PC compounds and the uses of CD-PMs-PC in biomedical sciences and demonstrated an outstanding enhancement, it is too soon to know if we understand proper usage with CD-PM-PC compounds.

Multidisciplinary strategies are expected to acquire additional details referring to PCs and their influence on nanomedicines to realize the favorite neurological and therapeutic consequences with nano-scaled materials. Solubility and production problems need to be considered before the application of CD-based theranostics in human treatment.

For the clinical translation of these prospectively favorable polymers, notable efforts must be taken in nano-processing, scale-up, and regulatory scientific work considering collaborative therapy and diagnostics. This is in addition to the latest clinical theranostic techniques. Using drug delivery techniques based on polymers and high molecular carriers CDs can enhance efficacy while minimizing the side effects. The excellent characteristics of CDs will offer opportunities for further development of theranostic nanomedicine methods. The appropriate assembly of CD-containing polymers can result in universally applicable polymers capable of serving in several fields, such as diagnosis, therapy, nanoscience, self-assembly, and cross-linked nanostructures. CDs make it possible to modify potent theranostic nano-medicines procedures to enhance treatment efficacy. Translation theranostics promotes multidisciplinary interaction between science and medicine to enhance disease research and drug development. The nano-bio interactions represent a major challenge in translating theranostic nono-medicine to clinics. The incompatibility or potential toxicity of bionanotechnology based on communication with natural material can cause disorders such as hyporesponsiveness, inflammation, or related diseases. The toxic influence is appreciably controlled by numerous parametric quantities like zeta potential, shape, and solubility of nano-formulations in solution [121].

Considering recent investigative capabilities, developed strategies will bring more advantages to increasing quality observation; this could be described as the interface between nanotechnology and biology. Overall, protein corona aspires to be a fascinating viewpoint tool in theranostics. In the last decade, medicine has moved to more informed healthcare management and treatment guided by theranostics. The search for markers for disease subtypes and more personalized treatment responses has been accelerated by academic scientists and pharmaceutical companies. About the power and wonderfulness of novel molecular technologies, the future of diagnostics (diagnostics, prognostics, and predictive) must reside in imaging for CD-based polymers. Imaging should diagnose the existence of a tumor, describe it, and monitor it relative to medical care. From the perspective of anatomical reference computed tomography (CT), magnetic resonance imaging (MRI) is the most accurate method, although these approaches cannot frequently differentiate between tumor and normal tissues. The regulative standards for these complicated polymers and their clinical trial results will reveal a model for future CD-based theranostic polymers.

Overall, these reports display novel approaches in the study of the theranostic nanomedicine field for superior management opportunities of diseases and their side effects. To our knowledge, no serious computational or experimental study has been reported on theranostic, nanomedicine, and CD-based polymers. Such a study can help improve the relevant strategies and provide the information for more in-depth studies. Considering recent analytical functionality, amended manners supply more occasions to boost quality declaration in theranostic nanomedicine.

## Figures and Tables

**Table 2:** Cyclodextrins for inhibition of Aβ fibrillation and chemical characterization methods.

Compound	CD Nature	Characterization Methods
Inhibition and/or disintegration of amyloid fibrils produced from human insulin and lysozyme proteins	Sulfobutylether (SBE7) β-CD	Steady-state and time-resolved fluorescence Circular Dichroism (CD) Fluorescence microscopic images
human islet amyloid polypeptide (hIAPP_20–29_): Cyclotriphosphazene (N_3_P_3_) and human islet amyloid polypeptide (hIAPP_20–29)_: α-cyclodextrin (αCD)	α-CD	Far-UV circular dichroism (CD) spectroscopy Fourier transformed infra-red (FTIR) spectroscopy Atomic force microscopy (AFM) Transmission electron microscopy (TEM) Identification of conjugated oligumers by size exclusion chromatography (SEC)

## Data Availability

Not applicable.
